# Supplementary material for: Ethylene Signaling Modulates Dehydrin Expression in Arabidopsis thaliana Under Prolonged Dehydration
Source: Int J Mol Sci. 2025 Apr 27;26(9):4148. doi: 10.3390/ijms26094148 (PMC12071766; doi:10.3390/ijms26094148)
Supplement: Supplementary file 1 [file ijms-26-04148-s001.zip › ijms-3528391-supplementary.pdf]

Ethylene Signaling Modulates Dehydrin Expression in *Arabidopsis thaliana* Under Prolonged Dehydration

Supplementary material

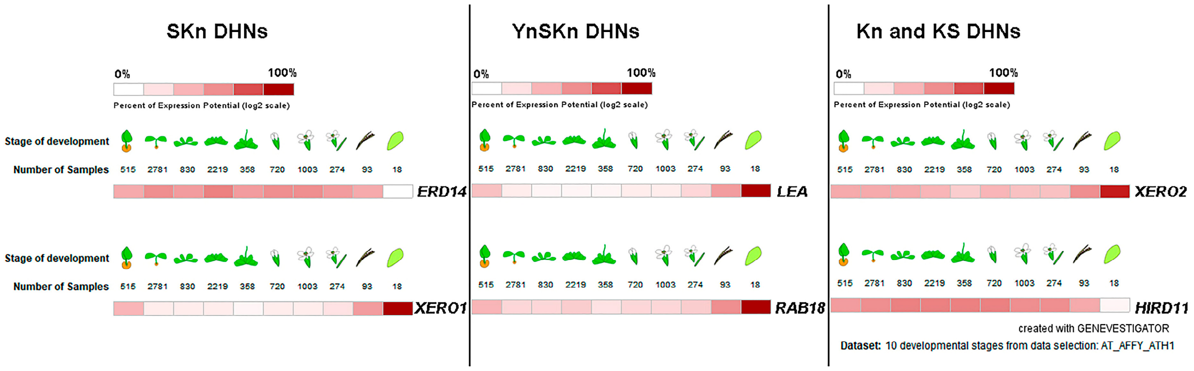

Figure S1. Expression profiles of six *Arabidopsis thaliana* dehydrin genes at different stages of ontogenesis generated by Genevestigator Plants (https://genevestigator.com/, site accessed on 03.11.2023)

DNH type	KS	Y3SK2	FSK3	FSK2	K5	SK2	FSK2	FSK3	K	Y2SK2
Gene ID	*HIRD11* AT1G54410	*LEA* AT2G21490	*ERD10* AT1G20450	*ERD14* AT1G76180	*XERO2* AT3G50970	*XERO1* AT3G50980	*DHN* AT4G38410	*COR47* AT1G20440	*DHN* AT4G39130	*RAB18* AT5G66400
**TF**	**Position vs. start of the coding region**									
**DREB19**	-962	**85**, -159, -290	-174, -967	-940	-573, -285, -193, -174, -101	-889, -664, -167, -160	-900, -848, -255, -155	-160, -773, -965	**17**, -974	-117, -285, -727, -990
**DREB1A**	-961	-160, -289	-165, -966	-802, -939	-572, -284, -194, -173, -102	-743, -168	-899, -849, -254, -239, -238, -156, -121, -120	-159, -964, -993	-149, -627, -628	-116, -209, -541, -690, -726
**DREB1E**	-960	-161, -288	**45**, -164, -965, -974	-435, -808, -938	-571, -367, -283, -195, -172, -103	-744, -169, -158	-850, -253, -157	-158, -992	-627, -628	-115, -208, -689, -926
**DREB1G**		-21, -161, -288, -345	-164, -843, -917, -937, -965	-938	-571, -223, -195, -172, -103, -98		-898, -850, -253, -157, -121, -120	-158, -913, -943, -963, -992	-627, -628	-115, -208, -283, -435, -540, -725, -926
**DREB26**		**87,** -295, -346, -560, -676	-43, -179	-10, -781	-575, -179	-965, -851	-905, -635, -472, -260, **6**	-4, -183, -487	**19,** -241, -979	-122, -534, -729
**DREB2**	-600	-289, -554	-173, -966	-7, -939	-572, -535, -194, -173, -102	-168, -116	-899, -849, -254, -156	-159, -964	-149, -628	-116, -209, -284, -690, -726
**DREB2C**		-288, -555, -567	-172, -937, -965	-938	-571, -283, -195, -172, -103	-975, -887, -858, -827, -744, -639, -169	-898, -850, -253, -157	-158, -963, -992	-972	-115, -283, -540, -725, -988
**RAV1**	-279	-163, -343, -559	-11, -512, -919	-729	-952, -819, -486, -308	-885, -746, -529, -508	-939, -852, -757, -443, -115	-284, -483, -530, -945	-78, -954, -968	-105, -113, -279, -433, -563, -877, -986
**RAV1_var.2**		-938	-910	**16**	-681, -521, -409	-239	-875, -378, -17	-713	-805, -862	-945
**RAP2-10**		-224, -275, -566	-173	-64, -775	-753, -574, -572	-974	-899, -121, -120, **90**		**16**, -76	-116, -284, -324, -511, -513, -726
**RAP26**		**84**	-176, -792	-61, -778	-575, -176	-971, -927, -663, -213	-944, -475, -257, -121, **90**	-62	**19**, -670, -973	-284, -510, -684, -726

Table S1. Position of DREB-binding sites in the promoter regions of dehydrin-coding genes of *Arabidopsis thaliana*, according to Eukaryotic Promoter Database, (https://epd.expasy.org/epd/, accessed on 18.12.2024). Grey color designates lack of binding positions for the particular TF and the pink color marks the presence of a binding position downstream TSS.

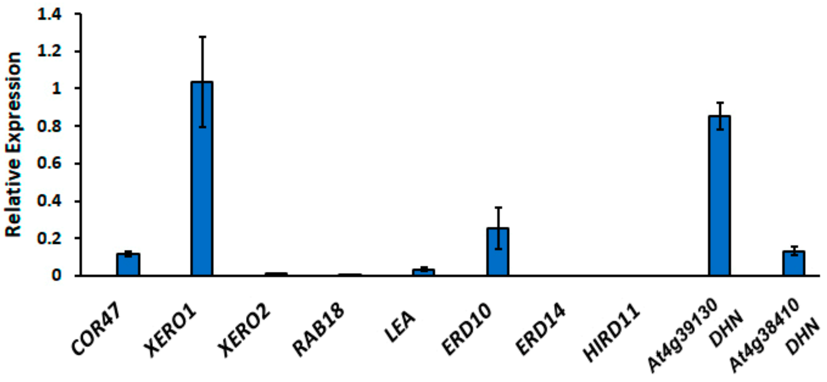

Figure S2. Relative expression of *A. thaliana* dehydrin genes in the wild type plants (Col-0) grown for 7 days on ½ MS media containing 2 μM of the ethylene precursor 1-aminocyclopropane carboxylic acid (ACC).

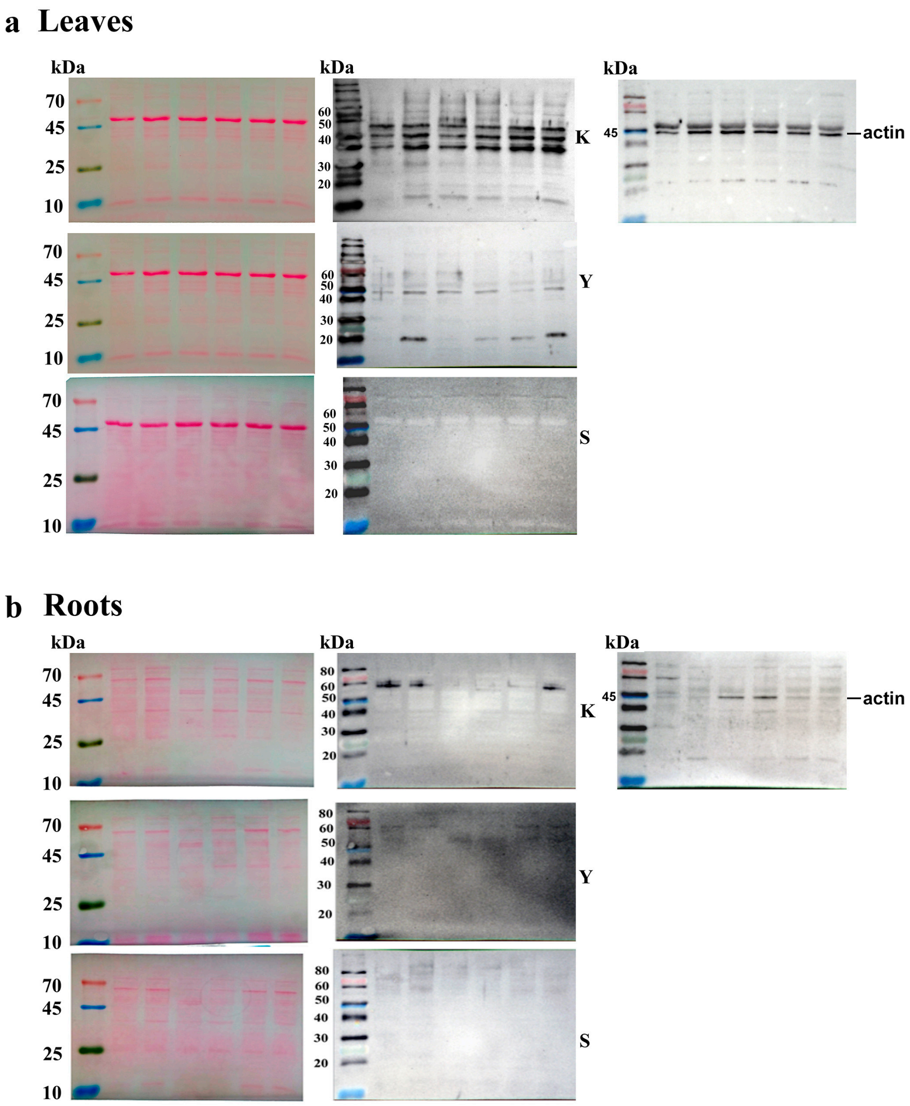

Figure S3. Ponceao S staining of the intact immunoblots used for the immunotetection of dehydrins in leaf (a) and root (b) samples of *A. thaliana* wild type (Col-0) and ethylene mutants (*ctr1-1* and *ein3eil1*). Monoclonal plant-specific anti-actin antibody (Sigma, Saint Louis, USA) was used to develop an internal protein reference on equally loaded membranes with leaf and root samples (on the right side of the figure panels).
